# Piloting an Intervention to Improve Outcomes in Young Adults Living With Type 1 Diabetes: The Experience of the D1 Now Support Worker

**DOI:** 10.3389/fcdhc.2021.799589

**Published:** 2021-01-28

**Authors:** Michelle Lowry, Eimear C. Morrissey, Sean F. Dinneen

**Affiliations:** ^1^ School of Medicine, National University of Ireland, Galway, Ireland; ^2^ Health Behaviour Change Research Group, School of Psychology, National University of Ireland, Galway, Ireland; ^3^ Centre for Diabetes, Endocrinology and Metabolism, Galway University Hospitals, Galway, Ireland

**Keywords:** young adults, type 1 diabetes (T1D), person centred care, diabetes distress, support worker

## Abstract

**Introduction:**

D1 Now is a novel intervention which aims to support self-management and clinic engagement and improve outcomes in young adults (18-25 years) living with type 1 diabetes in Ireland. It has been developed using a systematic, theoretical, user-centred approach. The specific role of the Support Worker, one of three components of the D1 Now intervention, was developed to provide continuity and build relationships between young adults and their diabetes team.

**Methods:**

A Support Worker - an Occupational Therapist, who had a background in youth mental health - was hired as part of the D1 Now pilot randomised controlled trial and was based in one intervention site to join the existing diabetes team.

**Discussion:**

The Support Worker aimed to provide an accessible and consistent point of contact for young adults, facilitated conversations about distress, and encouraged graded goal setting and collaborative problem solving. The role afforded her with a unique window into the lived experiences of young adults with type 1 diabetes where she observed the ongoing negotiation of life and living alongside diabetes care and management. The prevalence of diabetes distress was high in the study cohort with particular challenges associated with ‘all or nothing’ thinking patterns as well as disordered eating behaviours. The Support Worker also played an advocacy role in supporting the diabetes team’s awareness of young adults’ needs and explored current barriers to care. Preliminary findings from the D1 Now pilot have identified that the role of the Support Worker was viewed positively from the perspective of young adults with type 1 diabetes.

## Introduction

The life-stage of young or emerging adulthood is widely characterised as one of exploration and experimentation, featuring high levels of unpredictability and instability (1). Navigating the self-management of a demanding and complex condition such as type 1 diabetes (T1D) during this time poses significant challenges for those living with the condition. Traditional clinic-based models of diabetes care have struggled to meet the needs of young adults with T1D. A growing evidence base has demonstrated poor outcomes across a range of physiological, psychological and behavioural domains in this age group, including increased risk of suboptimal glycaemic control, higher incidence of diabetic ketoacidosis (DKA), disengagement from diabetes self-management including clinic attendance, as well as increased prevalence of psychological distress (2–7).

Diabetes distress is described as the emotional burden that people experience when living with diabetes and includes fluctuating levels of worries, concerns, fears and threats (8). Diabetes distress is noted to be relatively common, with approximately one third of adults with T1D reported to be living with high levels of distress at any one time (9). Mounting evidence suggests that it may be more prevalent than previously recognised among the young adult population due to the life-stage specific challenges which can compound T1D management (2). Opportunities to talk about diabetes distress with healthcare professionals (HCPs) was identified as a potential moderator of the distress experienced by young adults with T1D in a qualitative study by Balfe et al. (10). Additional research has highlighted that individuals are willing and value the opportunity to discuss the emotional aspects of their diabetes with their HCP (11, 12).

Professional guidelines and recommendations have long advised on the need for more holistic approaches to diabetes care and attention to the psychological aspects of diabetes (13–15). Despite this, it would appear that an implementation gap exists across diabetes care services with just half of diabetes HCPs, from a significant sample size of 4,785 HCPs surveyed across 17 countries, reported to provide psychological based assessment and support in practice (16, 17). Factors such as lack of time, privacy or comfort levels have been identified among the perceived barriers to discussing diabetes distress by both HCPs and those living with diabetes (18).

Over the past two decades, alongside greater consideration for the psychological aspects of diabetes care, diabetes guidelines and position statements have advocated for the adoption of person-centred approaches in diabetes care (15, 18). In the context of young adult diabetes care, a number of studies have pointed to the significant role of the relationship between young adults and their diabetes healthcare providers (19). A key finding by Hynes et al. (19) referred to the crucial role that positive clinic experiences, including interactions with service providers, has on the likelihood of young adults attending their clinic appointments.

## Background Context

**Figure 1:**
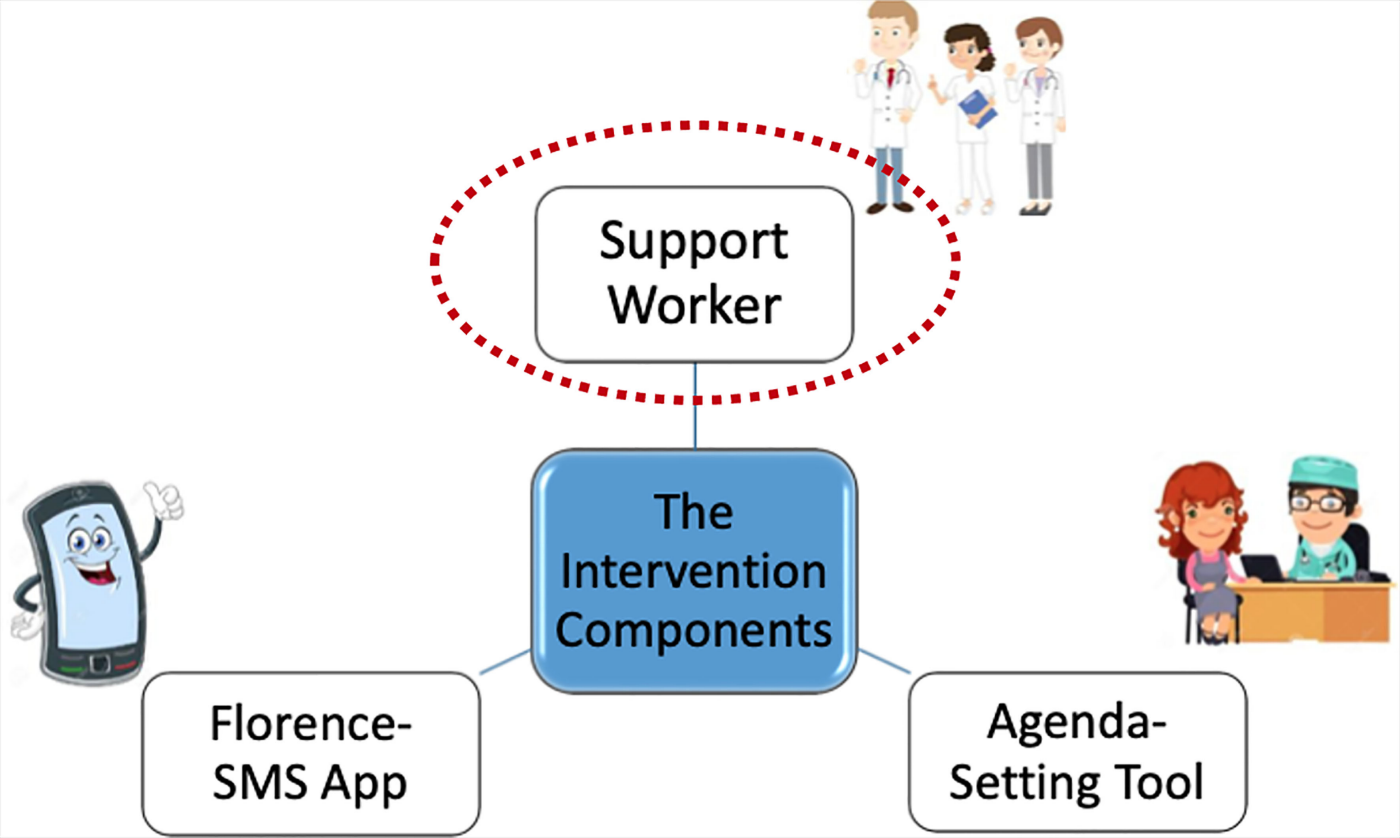
D1 Now intervention components.

In 2014, a multidisciplinary research team including a public and patient involvement (PPI) panel of young adults with lived experience of T1D (20), was established to develop a new intervention for young adults with T1D in Ireland, known as D1 Now. In Ireland, many hospital outpatient diabetes services offer dedicated ‘young adult’ clinics aimed at delivering care to individuals aged approximately 18-25. The D1 Now intervention is delivered as an adjunct to usual care within these young adult clinics. Details of the development process and the three intervention components: (i) a Support Worker; (ii) an interactive messaging system, ‘Florence’; and (iii) an Agenda-Setting Tool - have been described in a number of recent publications (see **Figure 1**) (21, 22). A pilot cluster randomised controlled trial (RCT) of the D1 Now intervention was carried out during 2019-2020 with the primary aim of gathering and analysing acceptability and feasibility data (23, 24).

The vision of the D1 Now study is to enhance young adult’s ability to self-manage their T1D and to improve a range of outcomes considered core to diabetes health and wellbeing (23). Each of the three components of the D1 Now intervention was designed as distinct but interrelated in their contribution to the study’s vision. The role of the Support Worker was intended to be additional to that of other members of the diabetes team - focusing on the provision of continuity and facilitation of relationship building and engagement between the young adult and their diabetes team (see **Supplementary Material** for job description) (23).

I was privileged to have had the opportunity to take up this role of Support Worker. My role was unique in that I was introduced as an external member to the diabetes team for the purpose of the study. My background as an Occupational Therapist (OT), and predominant experience in the area of mental health, meant that I was in relatively uncharted waters as a member of the diabetes team. Nevertheless, consistent with OT philosophy, I was well acquainted with the core values and delivery of person-centred practice which underpinned the role of the D1 Now Support Worker.

## Detail of Key Programmatic Elements

As part of the D1 Now intervention protocol, the Support Worker was required to be present at each young adult clinic appointment and ensure that the young adult has set an agenda for their appointment and that this agenda is followed through by the diabetes healthcare team. The Support Worker was intended to act as an advocate for the young adult on the clinic day and, if appropriate, contribute to multidisciplinary team discussions for each young person. In addition, the Support Worker was to communicate with the young adult between clinic appointments on an individual basis in order to support continuity of care.

## Discussion

### Young Adults With Type 1 Diabetes – An Insight Into the Lived Experience

Over the course of the study, I had the opportunity to work with over 20 young adults who provided me with a window into their experience of life with T1D. Maintaining a reflective journal throughout my experience in the role supported me to capture key learning from my work with these young adults. While each experience was individual in nature, there were a number of themes which stood out to me.

#### The Cost of Caring and Diabetes Distress Experienced by Young Adults

The prevalence of diabetes distress among young adults with whom I worked over the course of this study was striking. I was especially struck by the experiences of guilt and shame shared by many young adults who appeared to assume 100% responsibility for their diabetes – with little acknowledgement of the sheer complexity of the task at hand. High blood glucose (BG) numbers appeared to be received as judgements, a sign of failure, a stick with which they ought to beat themselves up with. A number of young adults described avoiding testing their BGs, not because they did not understand the importance of testing or that they did not care (a common stereotype that they had experienced), but rather in an attempt to avoid confronting the harsh criticism they have come to experience in the face of these dreaded numbers.

‘All or nothing’ thinking patterns appeared to present as a significant challenge for many young adults attempting to reach the somewhat elusive goalpost of ‘acceptable’ diabetes outcomes. Many appeared understandably disheartened by their HbA1c result (a blood test reflecting their level of glycaemic control) which did little to acknowledge the effort, challenges and compromises they have faced. Several young adults I met admitted to completely disengaging from their diabetes self-management at times, in an apparent attempt to avoid the persistent frustration they felt with the condition. Anecdotes of ‘*not knowing where to start’* and feeling overwhelmed by the constant struggle of integrating diabetes self-management tasks into current lifestyles were often identified as barriers to re-engaging in diabetes management.

While the experience of diabetes distress for the vast majority of young adults I met over the course of this study appeared to present at a ‘sub-clinical’ level, there were a number of individuals for whom diabetes distress was associated with a co-morbid diagnosis of an eating disorder (ED). EDs are serious and complex mental health issues – and can involve a range of disordered eating and weight control behaviours, which in the case of T1D can include deliberate insulin manipulation. The prevalence and complexity of ED in the context of T1D was not something I was overly familiar with before the study. It would appear that despite the known prevalence and clinical significance of EDs in T1D, current services, both at home and abroad, are struggling to adequately meet the needs of those presenting with these challenges (25). What I have learned from the young adults I worked with is that some of the basic tenets of T1D management can completely conflict with those of ED treatment. Basic routine clinic practices of weighing young adults at each clinic visit presented as a significant challenge and requests to avoid being weighed were not always appropriately managed. Recommendations to closely monitor foods and count carbohydrates appeared to conflict with goals of reducing ‘rule-driven’ eating. Expectations to share their BG data with unfamiliar health professionals was perceived as exceptionally intrusive, particularly as EDs are renowned to thrive on secrecy and shame (26). The quality of the relationship between young adults and diabetes team members seemed crucial in ensuring individual needs could be understood and the appropriate level of support provided. Young adults I met described finding it helpful when diabetes teams and ED support services shared communication and their care felt coordinated, however it was noted that this was not always their experience.

#### Navigating Life and Fitting Diabetes In

Diabetes was often regarded at best as a major inconvenience (there were more colourful descriptions)! for the young adults with whom I worked. Life appeared fast-paced, varied and ever-changing. Having a predictable routine seemed rare for most. Many young adults were balancing employment alongside education, navigating increased independence while still living at home, forming new friendships while maintaining old ones, exploring romantic relationships, new interests and hobbies. As is typically associated with the life-stage of emerging adulthood, many appeared to be attempting to make sense of who they are and who they wanted to be in this very uncertain world which brought with it a lot of pressure (1). Diabetes formed one part of a much bigger picture and from what I could gather from my conversations with this group – the ultimate goal was to be able to get on with the ‘life stuff’ and for diabetes to cause as little disruption as possible.

It would appear that the current thinking in diabetes care is that through the provision of education, people with diabetes ‘should’ be able to self-manage their condition well (27). While education has undoubtedly a vital role in supporting diabetes self-management, it is imperative to acknowledge the complexity of the task for young adults. Consistent with findings by Hynes et al. (19) - there appeared to be attempts among young adults to ‘function as normal’ – like their peers, colleagues and classmates without T1D. A number of individuals described certain workplaces taking issue with the time required to engage in diabetes self-management tasks and the hesitancy they felt in requesting designated breaks. Many described intentionally running BGs high in an attempt to avoid a hypoglycaemic event at work or in college. This fear of hypoglycaemia and the associated disruption of the experience appeared to resonate with many of the young adults across a number of valued life areas including college/work but also in social contexts and their leisure pursuits which included competitive sports. The immediacy of the benefits associated with this ability to ‘get on with life’ afforded by running BGs high appeared to outweigh potential longer-term costs to health – however the majority still appeared acutely aware of the implications of such decisions and carried an unresolved guilt.

Diabetes care did not always appear to integrate well into young adult’s lives – with one young adult describing her wish to ‘*just get in – get out’* of her clinic appointment so that she could get back to her life. The introduction of diabetes technology appeared to be a game-changer for some young adults, however the role of the diabetes team as gatekeepers to accessing new technology posed certain challenges. The expectation to attend various different clinic appointments, endure lengthy waiting times or join week-long education groups appeared unrealistic for some.

### So What Exactly Was the Role of the Support Worker?

I spent the initial few weeks in the role really trying to figure out how best I might be able to support the young adults who had agreed to participate in the study. I was excited but apprehensive about the potential scope of the role. I’ll admit there were initial grand plans of coffee mornings, group activities and intensive goal-setting sessions (that may have been the OT in me). What I soon realised was that each individual would be my guide – and my main role was to integrate into, and support rather than further disrupt the lives of these young adults.

#### An Accessible Point of Contact

Above all else for these young adults, I was to be an accessible point of contact who they knew they could contact if and when they needed to. As per the research protocol, I was present for all clinic appointments so became a familiar face with whom they could expect to meet at each clinic visit. These encounters proved important in affording the opportunity to build relationships. Outside of clinic appointments, the platform of WhatsApp appeared to be the most desired medium through which contact was exchanged. Others opted to use SMS or email. The days and hours I would be contactable was made clear to young adults who appeared satisfied to be able to make contact at a time that worked for them and receive their response at some point during my working schedule.

Looking back over the communications shared – exchanges between myself and young adults included informal check-ins between clinic visits to follow up on identified goal areas, sharing relevant information resources, queries and reminders related to upcoming appointments, and relaying information between young adults and the team e.g. update on a continuous glucose monitor (CGM) application. It is important to highlight that the level of contact and engagement between myself and young adults in the study varied significantly across individuals – with some having quite regular contact between clinic visits and others none at all. In the beginning, I noted that I was often the one to initiate a check-in, however as the study progressed, and relationships developed, I was conscious to try to follow the lead of each individual in terms of responding to specific requests or previously agreed action areas.

#### Facilitated Conversations About Distress

One of my main roles on clinic day was to support young adults in their completion of the Agenda Setting Tool - another D1 Now intervention component - in preparation for their conversation with the doctor. The Agenda Setting Tool aimed to support young adults in identifying what they themselves would like to get out of their time in clinic. The tool also included two specific questions related to diabetes distress [DDS-2; (28)] which served as a brief screening tool and provided the opportunity to explore experiences of distress further [option to administer the full Type 1 Diabetes Distress Scale with 28 items; (29)]. As previously noted, the prevalence of distress among young adults could not be ignored. It was apparent from my interactions, and indeed consistent with current literature, that being asked questions about experiences of distress was relatively new for the majority of these young adults.

There are a number of reported barriers to addressing issues of distress within traditional models of diabetes care such as lack of time and confidence as well as fears associated with the dreaded ‘can of worms’ which may be unleashed (30). But like it or not, the so-called ‘worms’ are there and denying or ignoring their presence does little to contain or manage the negative impact they are already having on so many individuals we as HCPs are in a position to support.

While conscious of the notable dearth of dedicated psychology resources within the diabetes service, I was reassured by the direct experience of engaging young adults in conversations about distress. For the vast majority, it seemed that having the opportunity to acknowledge experiences of distress, and to feel validated and reassured seemed to be helpful in mitigating the impact of diabetes distress. While there will always be some individuals with certain needs warranting more specialist support (and for those we need to continue to advocate for appropriate service provision and coordinated care), it is imperative that we are effectively using the current resources we have available to sustain collective efforts ‘upstream’ to support not only the physical aspects but also the emotional aspects for all living with T1D.

These resources do not necessitate ‘specialist training’ but speak more to the core values of person-centredness and include interpersonal skills of conveying empathy and actively listening. Through conversation, there was the opportunity to better understand specific sources of distress and explore the most appropriate support options. For some, this support came from within the service and included accessing practical guidance on adjusting treatment regimens or applying for a CGM to reduce the burden of BG testing. For others, these conversations often shone a light on unhelpful thinking styles which appeared to be exacerbating their distress (*“I’m lazy”, “I’m letting everyone down*”). There were times when the sources of distress were external to diabetes (e.g., relationship break-up, conflict at work, exam pressure) but nevertheless were having a knock-on impact on the young adult’s ability to self-manage well. While I may not have always been in a position to help resolve sources of distress, providing a platform which acknowledged that we cannot separate our physical from our emotional health or indeed our diabetes from life – seemed to be of value to the young adults with whom I worked.

#### Graded Goal Setting and Collaborative Problem Solving

Over the course of the study, I availed of opportunities to explore the costs of ‘all or nothing’ thinking traps and tried to facilitate a shift in focus to process (actions) rather than outcome (numbers). This shift in focus also allowed individuals to ‘zoom out’ somewhat and connect with what matters to them most – diabetes health repositioned as a resource to but not the objective of life. Borrowed from Acceptance and Commitment therapy (ACT), the concept of ‘towards moves’ was introduced to support the setting of small but achievable goals and also allowed for flexibility to adjust to the inevitable challenges both life and diabetes presented.

Acknowledging anticipated barriers in pursuit of goal areas was a sometimes challenging but valuable conversation. It was important to identify the existing resources available to young adults during these conversations, providing a platform to reflect on how they themselves have managed challenges in the past, what has and has not worked well for them rather than prescribing generic ‘solutions’. The young adults I met were often their own best resource – the experience of navigating life with T1D having already provided them with an invaluable skillset that should not be underestimated. Exploring expectations of what is ‘good enough’ rather than ‘perfect’ appeared helpful and facilitated greater flexibility (and potential sustainability) in their approach to diabetes self-management.

#### Advocacy and Empowerment

The Support Worker role afforded me with the opportunity to build relationships with young adults participating in the study, develop an understanding of their individual life situation and an awareness of how diabetes currently integrates into this picture. I was able to explore the potential barriers and challenges individuals may be facing in accessing various aspects of diabetes care. While our health system strives to ensure equality in terms of policies, procedures and service provision – it became apparent to me that this does not always ensure equity of access for all individuals, and there are significant gaps as a result.

Working with this group of young adults, it was clear that certain individuals did not always have access to the information they needed in order to make informed decisions about their diabetes care. Some rejected invites to attend structured education courses often unclear of the purpose or value of the course. Others were unaware that such courses even existed. While for certain individuals, the time commitment required, or the group context of the learning space presented as a significant barrier. One individual I worked with spoke of their receptive communication difficulties which contributed to significant challenges in processing and recalling verbal information (which they found was often the predominant mode of communication during clinic encounters). Providing platforms to explore perceived barriers to accessing education rather than assuming individuals are ‘not interested’ or ‘unmotivated’ was valuable and created the opportunity to collaboratively identify ways of overcoming such barriers (for e.g.: developing youth-friendly information resources on the ‘why and what’ of structured education, negotiating 1-1 sessions with dietitian or diabetes nurse specialist, signposting to online self-directed education courses). The D1 Now PPI panel who were co-researchers on this study were an invaluable support in co-creating various resources which could be shared with young adults during the study and are now also freely accessible online (see: https://d1now.ie/blogs/articles).

Inequity challenges also presented in terms of access to new technologies. There appeared to be a ‘fight’ to access new technology for certain individuals, and those who were well-informed and able to articulate themselves well seemed to fair best in these ‘battles’. Eligibility criteria for diabetes technologies appeared to lack sufficient transparency and there were notable inconsistencies in terms of the approval process which contributed to shared frustrations for both young adults and HCPs alike. I became acutely aware of the complex power dynamic at play in the context of accessing diabetes technologies – particularly given the range of stakeholders involved (i.e. young adult with diabetes, diabetes HCPs, funders and policy makers). Such experiences are consistent with recent research findings by Gajewska et al. (31) who has highlighted gaps in the delivery of diabetes care in Ireland which are believed to contribute to the barriers experienced in accessing technology. Gajewska et al. (31) advocates for the need to reduce the disparities in current health service provision. In my role as Support Worker, I found myself supporting young adults in preparing for conversations around technology requests and justifying their needs in the context of their life circumstances. As I reflect on the experience, I am curious about the young adult voices we do not always hear and who may perhaps be left behind.

### Key Learning – My Take Home Messages From My Experience as Support Worker

- Diabetes management is a lifelong endeavour – a marathon not a sprint. Considering the individual life context within which diabetes self-management happens is crucial. Developing collaborative relationships with young adults within a culture of care, empathy and respect is a worthwhile investment.
- While the rhetoric of person-centred care and empowerment-based approaches in diabetes care is strong, we need continued effort to close the implementation gap in terms of the realisation of these core principles into everyday practice. Asking a young person *‘what they would like to talk about in their clinic appointment’* appeared to be a significant starting point.
- We cannot ignore issues of diabetes distress! It is crucial that we are providing the opportunity to discuss issues of distress as part of routine care. We should not underestimate the value of listening and validating these experiences. *Brief screening tools such as the DDS-2 are easy to use and provide a great opportunity to open up this conversation.*
- Diabetes care may need to look differently for different people in order to ensure equity of access for all. Our current health system may need to be further challenged in order to support diabetes HCPs to offer a more flexible and responsive model of care – ‘*care that fits*’ young adults and their individual needs (32).

### Acknowledgement of Any Conceptual or Methodological Constraints

As with the introduction of any new role to an established team, particularly one which has not yet been fully defined (as was the case with the D1 Now Support Worker), time and opportunities were needed to build collaborative relationships with existing members of the diabetes team. In the time-limited context of a pilot RCT, this proved to be somewhat challenging. In many ways, the D1 Now Support Worker represented a change from traditional practice so navigating the boundaries of the role and ensuring a collaborative approach was crucial if the young adults attending the clinic were to fully benefit from the intervention received.

The global pandemic of COVID-19 occurred mid-pilot RCT which resulted in clinics being moved to a virtual environment for at least three months in participating centres. The D1 Now intervention was originally designed for an in-person clinic, and it is possible that participants did not receive the full intervention experience during this time. Nevertheless, flexibility afforded by the Support Worker role meant that communication and maintenance of relationships with young adult participants could be supported through virtual channels of video calls, instant-messaging and email exchanges.

Although findings from the D1 Now pilot RCT indicate that the Support Worker was perceived by both young adults and healthcare professionals as a valued addition to the diabetes team, there were concerns shared about how a Support Worker would be resourced outside of a research study context. Current literature related to the potential role of allied health professionals such as Occupational Therapy in diabetes care is really only just emerging. Recent research studies across the UK and USA demonstrate the valuable contribution OT can make to a range of diabetes populations, across the lifespan and in a range of settings (33–36). OT practice in Ireland appears slow to build on this work of international colleagues. Although the job description (see **Supplementary Material**) and eligibility criteria for the D1 Now ‘Support Worker’ was open to a range of professional backgrounds including nursing, psychology and allied health, I believe, albeit with some level of bias, that there are obvious parallels between the role of Support Worker and that of OT. OT is a profession which aims to enable individuals to maximise their capacity to participate in life activities which are both important and meaningful to them (37). OT views the person in the context of their individual life circumstances, rather than focusing on their health condition. Collaborative goal setting, problem-solving and the development of coping skills are all central elements of OT interventions and featured heavily in the interventions I facilitated as D1 Now Support Worker. Perhaps there is a case to be made for Occupational Therapy establishing its role as a core member of the diabetes team here in Ireland.

## Data Availability Statement

The original contributions presented in the study are included in the article/**Supplementary Material**. Further inquiries can be directed to the corresponding author.

## Ethics Statement

The studies involving human participants were reviewed and approved by Beaumont Hospital Ethics Committee (ref: 19/51). The patients/participants provided their written informed consent to participate in this study.

## Author Contributions

SD co-conceived the study idea. EM managed the study implementation. EM and ML contributed to data collection. ML drafted the manuscript. EM, SD, and ML revised the manuscript. All authors read and approved the final manuscript.

## Funding

This work has been funded by a Definitive Intervention and Feasibility Award from the Health Research Board in Ireland (DIFA-2017-034).

## Conflict of Interest

The authors declare that the research was conducted in the absence of any commercial or financial relationships that could be construed as a potential conflict of interest.

## Publisher’s Note

All claims expressed in this article are solely those of the authors and do not necessarily represent those of their affiliated organizations, or those of the publisher, the editors and the reviewers. Any product that may be evaluated in this article, or claim that may be made by its manufacturer, is not guaranteed or endorsed by the publisher.
