# Supplementary material for: Piloting an Intervention to Improve Outcomes in Young Adults Living With Type 1 Diabetes: The Experience of the D1 Now Support Worker
Source: Front Clin Diabetes Healthc. 2021 Jan 28;2:799589. doi: 10.3389/fcdhc.2021.799589 (PMC10012156; doi:10.3389/fcdhc.2021.799589)
Supplement: Supplementary file 1 [file Table_1.docx]

**D1 Now Support Worker**

**Job Description:**

The Support Worker role is an expansion of the Diabetes team. The Support Worker has a specific focus on supporting young adults with Type 1 Diabetes and will act as an advocate for these young adults to ensure optimal engagement with the Diabetes service. The successful candidate will work closely with the young adults and their respective Diabetes team (doctors, nurses, allied health care professionals, psychology, etc.).

**Duties/ Key Responsibilities:**

- To work closely with the chosen Diabetes clinics and members of the D1 Now research team.
- To be a voice/advocate for the young adult through attendance at multidisciplinary meetings within the Diabetes clinic.
- To provide consistency for the young adult by regular attendance at clinics and having a well-rounded knowledge of the young adults medical and social history.
- To communicate with the young adult between their clinical appointments to follow-up on any tasks required and monitor progress with self-management strategies/techniques.
- To manage expectations of the Diabetes service and to conduct a needs/priority assessment for the young adult.
- To assist in organising/rescheduling appointments at the clinic.
- To refer the young adult to structured education, psychology or other services within the diabetes clinic though communication with the multidisciplinary team.
- To work closely with the D1-Now intervention team and additional D1 Now components including an interactive online tool for self-management and an agenda setting tool for use in clinic appointments.
- To be flexible in communicating with the young adult in a suitable location (hospital or community setting) and in a suitable medium (face-face meeting, phone, text, group, etc.)
- To aid relationship building between the young adult and their Diabetes team.
- To carry out administrative duties as required by the post.
- To share information with the Diabetes team and D1-Now team and other services as appropriate in accordance with good practice.
- To be flexible regarding working hours in line with the needs of the role (incorporating occasional late evening work, etc.).
- To participate in education, training and networking events as appropriate

**Qualifications/Skills required:**

**Essential Requirements:**

- A recognised professional qualification in Nursing, Mental Health Nursing, Psychology* (postgraduate qualification in Clinical/Counselling/Educational Psychology/Health Psychology), Social Work or Allied Health Care.

*Assistant Psychology posts will be provided supervision by a Clinical Psychologist.

- Experience of working with clinical populations, ideally young people or people with long-term conditions, in a clinical setting (hospital or community).
- Well-developed communication skills and an ability to interact with both young people and adults on a one to one basis and in small groups, within a range of contexts.
- Confident lone working with the young adult patient.
- Ability to plan and manage own workload.
- Highly motivated and a passion for working with young adults.
- Willing to be flexible and work evenings where required.
- Proficiency with computer systems.

**Desirable Requirements:**

- Experience of working with people with Diabetes or young adults with long-term conditions.
- An understanding of diabetes and the issues faced by young people as they transition to adult services.
- Knowledge and understanding of the physical, social and emotional developmental needs of young people.
- Experience of working in a multidisciplinary team in the hospital setting in Ireland.
- Some knowledge and/or experience of motivational interviewing or appropriate behavioural training.
